# Lipoma of the nasal septum: A case report

**DOI:** 10.1002/ccr3.3359

**Published:** 2020-09-16

**Authors:** Hesam Jahandideh, Fatemeh Dehghani Firouzabadi, Mohammad Dehghani Firouzabadi, Delaram Jan, Maryam Roomiani

**Affiliations:** ^1^ ENT and Head & Neck Research Center The Five Senses Institute Iran University of Medical Sciences Tehran Iran; ^2^ Department of Otolaryngology‐Head and Neck Surgery Firoozgar Hospital Iran University of Medical Sciences Tehran Iran

**Keywords:** case report, lipoma, nasal obstruction, nasal septum, nasopharynx mass

## Abstract

Even routine diagnoses, such as septal deviation, which most people do not think need imaging, require careful examination because rare diagnoses such as lipoma may occur in the nose. Careful examination and imaging lead to the best treatment.

## Graphical abstract

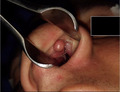

## 1 INTRODUCTION

A 22‐year‐old man presented to our hospital with the chief complaint of nasal obstruction. Computed tomography (CT) without contrast revealed unusual septal thickness, and subsequent magnetic resonance imaging showed an amorphous heterogenous fat content mass. The mass was excised using submucoperichondrial approach with no complication.

The lipoma is the most common soft tissue neoplasm in adults. Its highest incidence is among those aged 40‐50 years and is slightly more prevalent in males. It accounts for differential diagnosis of nasopharynx mass as a benign one that, in most cases, is located in the upper back, shoulders, arms, buttocks, and upper thigh.^1^ The prevalence of this painless tumor is one percent in the general population and is not common in the midface, and it rarely occurs in paranasal sinuses or nasal cavity due to the scarce amount of fatty tissue.^2^ Here, we have reported a rare case of nasal lipoma, which is found on the nasal septum.

## 2 CASE PRESENTATION

**Figure 1:**
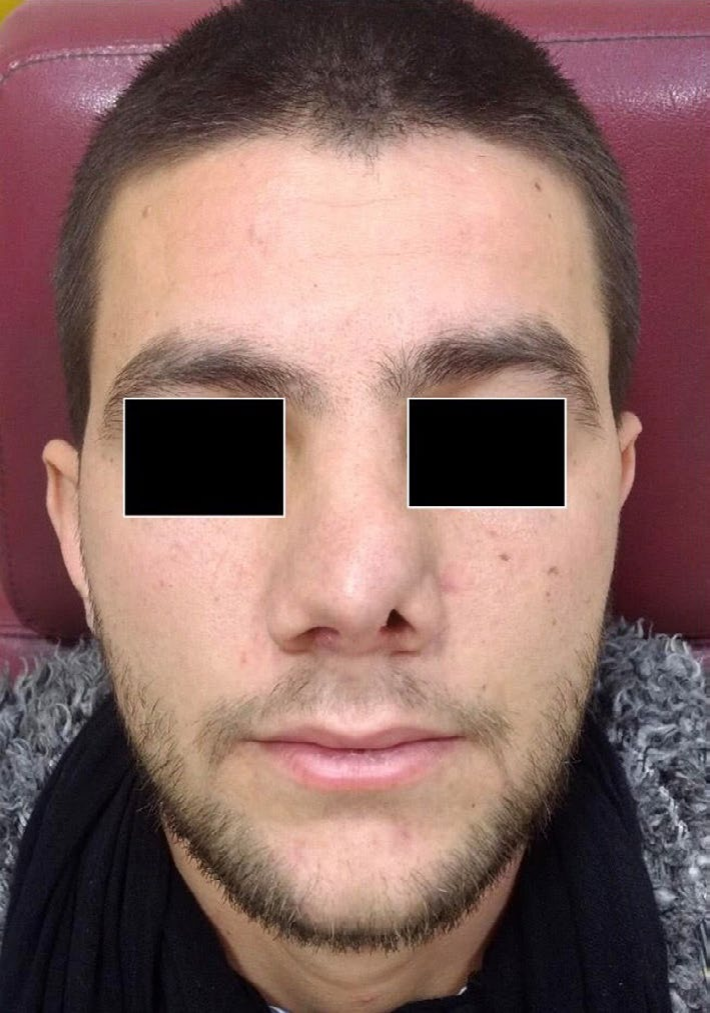
Nasal deformity and deviation due to underlying septal mass

**Figure 2:**
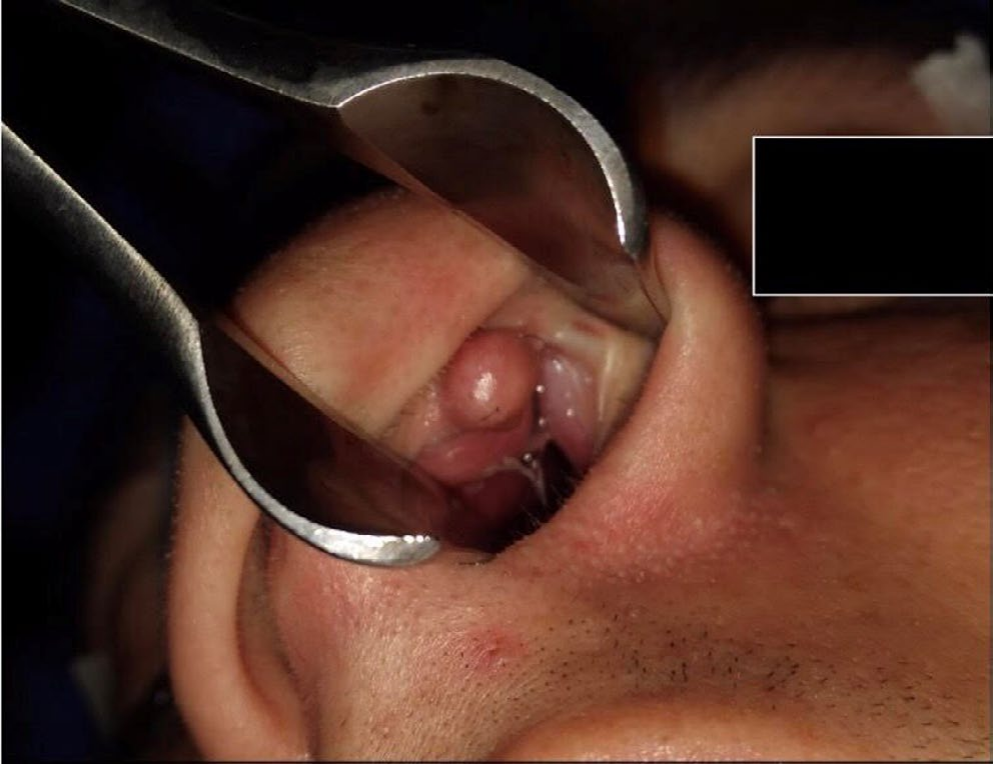
More careful inspection of upper part of setup revealed a round mass

**Figure 3:**
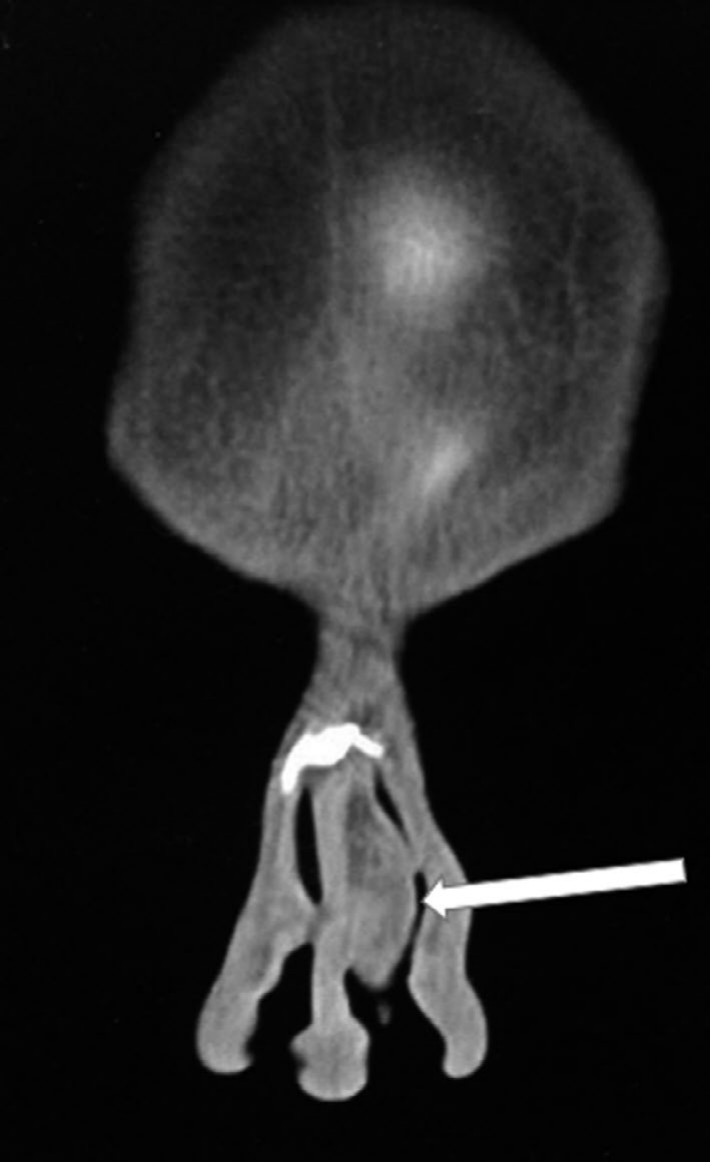
Hypodense mass 54*8*94 is nasal septum

**Figure 4:**
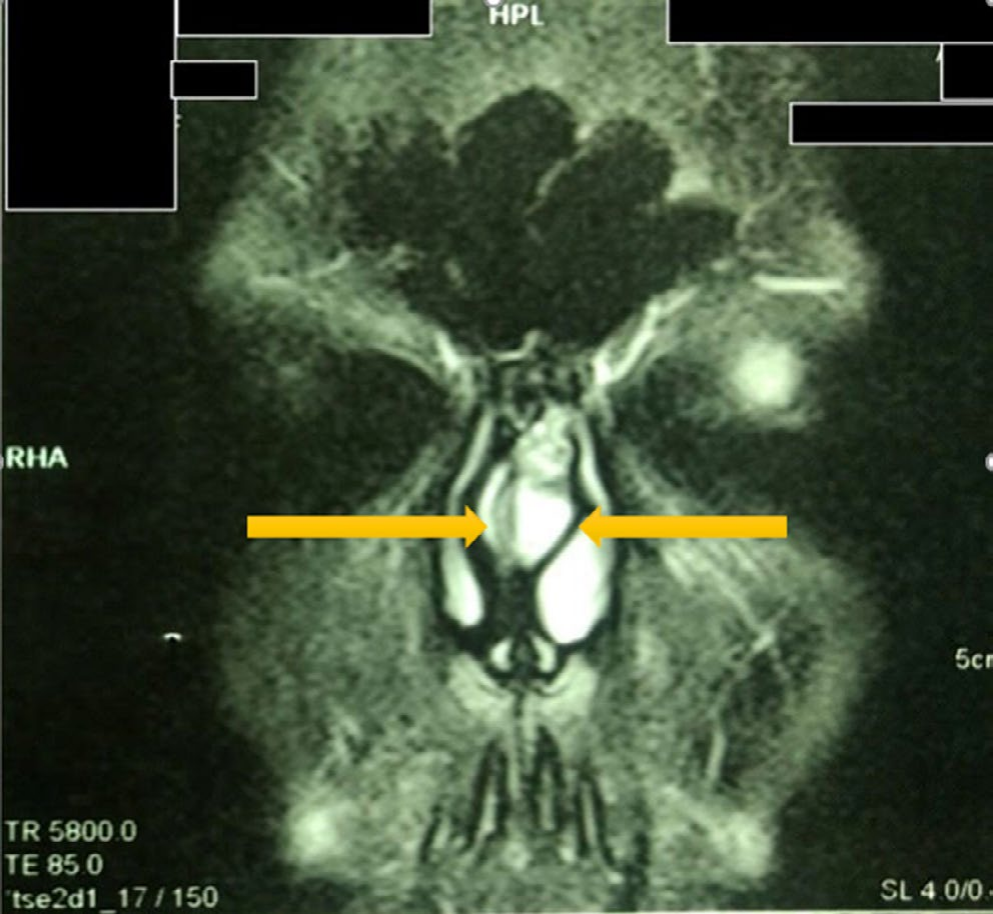
T2‐weighted MRI shows bizarre‐shaped 54*8*94 mm hyper signal mass located in left anterior part of the nasal septum

**Figure 5:**
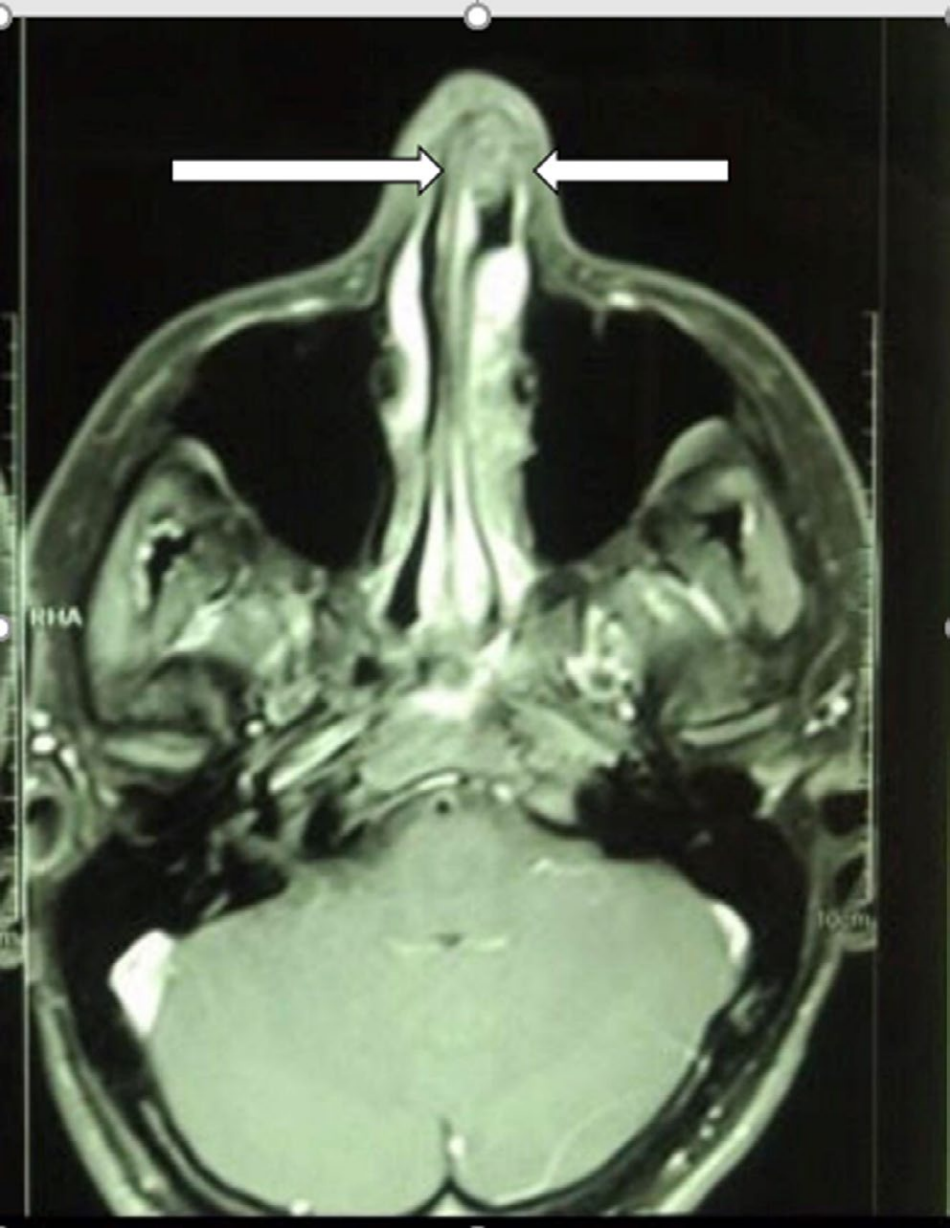
IV contrast T1‐weighted MRI. nonenhancing mass in left anterior part of the nasal septum

A 22‐year‐old man presented to our hospital with left‐sided nasal obstruction for more than 2 years. The patient's medical history, including the previous history of allergy or surgery, was nonspecific. In physical examination, left‐sided septal bulging, which was soft and compressible, was observed (Figure 1). A more careful inspection of the upper part of the septum revealed a distinct small round mass (Figure 2). CT scan without contrast revealed a hypodense mass with 1.5 cm thickness (Figure 3). Further evaluation with magnetic resonance imaging (MRI) to inspect any possible intracranial connection showed an amorphous heterogenous 54*8*94 mm mass located in the left anterior part of the nasal septum. The mass was a hyper signal in T2‐weighted images and did not enhance after gadolinium injection, contrary to its overlying normal nasal mucosa (Figures 4 and 5). An incisional biopsy was performed on the mass, and the homogenous yellow specimen was reported lipoma. On histopathological examination, the left nasal cavity lesion excision showed a piece of creamy, firm tissue. Under general anesthesia, the mucoperichondrial flap was elevated using left hemitransfixion incision, and the tumor was excised completely. Histopathology report confirmed lipoma. The patient presented no complications during or after surgery. The patient was followed up for 6 months. His nasal obstruction was improved significantly, and the apparent nasal deviation was alleviated to some degree. No recurrence of the tumor was detected in the follow‐up period.

## 3 DISCUSSION

Lipoma is a benign slow‐growing neoplasm that mostly consists of mature adapoid cells. Thirteen percent of lipomas occur in head and neck, which the posterior neck, chicks, tongue, floor of the mouth, and buccal sulcus are the most common.^3^, ^4^ Lipoma of the nasal cavity is rare and is mostly reported in children as a single mass or a piece of a syndrome.^5^, ^6^, ^7^ There are few reports of lipomas in different parts of the nose, including nasopharynx, vestibule, nasal dorsum, and inferior turbinate.^4^, ^8^, ^9^, ^10^, ^11^, ^12^ Patients may be asymptomatic, or like other masses occurring in sinuses and nasal cavity, they may present with symptoms, including nasal obstruction, facial edema, tenderness, rupture, and hemorrhage.^8^, ^13^, ^14^, ^15^, ^16^, ^17^

There have been reports of pediatric nasal lipoma and their associations with midline facial defects and different syndromes.^18^ Pai syndrome has been described primarily in 1987 as a condition consisting of congenital nasal lipoma, midline cleft of upper lip, skin and nasal polyps, and lipoma of the central nervous system.^6^, ^19^ Other reports indicated the nonsyndromic association of nasal lipoma and intracranial lipoma, especially in the corpus callosum.^15^ In adults, however, the first case of nasal septal lipoma has been reported in 2000 in a 21 years old woman referring with unilateral nasal obstruction and posterior septal soft tissue mass.^4^ CT scan can reveal a homogenous mostly not encapsulated low‐density mass, facilitating the diagnosis by showing the characteristics of fat tissue.^20^ It can also help in finding the possible intracranial extension and boney midline defect of the face and skull base. Some authors suggested using CT in all cases of pediatric midline nasal lipomas.^5^

In our study, nasal septal lipoma was reported in a 22‐year‐old man. Tumor removal was done using the mucoperichondrial flap. Differential diagnoses of nasal lipoma are dermoid cysts, teratoma, glioma, encephalocele, and meningomyelocele, which all of them may resemble a deviation if the septum is examined in less than a meticulous way^18^

## 4 CONCLUSION

Lipoma of the nasal septum is a rare presentation. We reported a case of nasal septal lipoma, presented with a nasal obstruction that could easily pass as a septal deviation without a conscientious examination. Performing imaging studies before septoplasty in cases of nasal obstruction with questionable physical examination could lead to a precise diagnosis.

## CONFLICT OF INTEREST

Authors declare no conflict of interest.

## AUTHOR'S CONTRIBUTIONS

HJ, MR, and FDF: conceptualized and designed the work. HJ, FDF, MDF, DJ, and MR: critically revised the article. All authors: approved the final version and have the agreement to be accountable for all aspects of the work in ensuring that questions related to the accuracy or integrity of any part of the work are appropriately investigated and resolved.

## ETHICAL APPROVAL

This study protocol was approved by the local ethics committee of the Iran University of Medical Sciences. Informed consent was obtained from the patient before the study.
